# Quantitatively evaluating the effect of social barriers: a case–control study of family members’ opposition and women’s intention to use contraception in Pakistan

**DOI:** 10.1186/s12982-015-0023-x

**Published:** 2015-01-24

**Authors:** Mishal S Khan, Farah Naz Hashmani, Owais Ahmed, Minaal Khan, Sajjad Ahmed, Shershah Syed, Fahad Qazi

**Affiliations:** London School of Hygiene and Tropical Medicine, London, UK; Research Alliance for Advocacy and Development, Aga Khan III Road, Karachi, Pakistan; Imperial College, London, UK; Pakistan National Forum on Women’s Health, Karachi, Pakistan

**Keywords:** Case–control, Family planning, Family opposition, Pakistan

## Abstract

**Background:**

Uptake of family planning services in Pakistan has remained slow over the past decade despite a rapid increase in availability and awareness, indicating that social barriers may be preventing uptake. Social barriers such as opposition by family members have largely been studied qualitatively; there is a lack of quantitative evidence about the effect of different family members’ opposition on women’s intention to use contraceptives. The objective of this study was to quantitatively evaluate the effect of family members’ opposition to family planning on intention to use contraception amongst poor women in Pakistan who have physical access to family planning services.

**Methods:**

An unmatched case control study (nested within a larger cohort study) was conducted in two public hospitals in Karachi, Pakistan. Univariable and multivariable logistic regression analyses were conducted to compare risk factors between women that were not intending to use any contraceptive methods in the future (cases) and women that were planning to use contraceptive methods (controls).

**Results:**

248 cases and 496 controls were included in the study. Negative contraceptive intent was associated with no knowledge of contraception (AOR = 3.79 [2.43-5.90]; p < 0.001), husband’s opposition (AOR = 21.87 [13.21-36.21]; p < 0.001) and mother-in-law’s opposition (AOR = 4.06 [1.77-9.30]; p < 0.001).

**Conclusions:**

This study is the first to quantitatively assess the effect of opposition by different family members on women’s contraceptive intent in Pakistan. Our results indicate that of all family members, husband’s opposition has the strongest effect on women’s intention to use contraception, even when the women have knowledge of and physical access to family planning services.

## Background

Pakistan is globally recognised as a country facing challenges in controlling its population growth. The birth rate has remained higher and the contraceptive prevalence rate lower than in neighbouring countries with similar or worse socio-economic conditions [1]. The Pakistan Demographic and Health survey conducted in 2006–7 found that only 30% of married women use contraception [2].

Most quantitative studies on family planning (FP) in Pakistan analyse secondary data from national surveys, and investigate distal determinants of contraceptive use such as wealth, education and rural versus urban residence. These studies have reported that proximity to community health workers and schools, urban residence, greater wealth, exposure to family planning messages in the media, and women’s level of education are positively associated with contraceptive use [3,4]. Based on the observation that uptake of FP services in Pakistan has increased very slowly over the past decade despite a rapid increase in availability and awareness [4], some researchers suggest that women’s demand for and access to contraception is not translating into contraceptive use due to social barriers from family members [5]. This is supported by the limited literature available on this topic, which is largely based on qualitative studies.

Qualitative studies have shown that a major factor influencing Pakistani women’s intention to use any methods of contraception is the permission/opinion of key decision-makers, particularly husbands [6,7]. Analyses of survey data to investigate more proximal influences on women’s decision-making have also found that factors such as agreement with husband on the ideal number of children and perceived support from in-laws are positively associated with intention to use contraceptives [8,9]. Despite consistent evidence emerging that family members’ opposition to FP has a major influence on women’s decisions about contraception [10,11], there is a lack of quantitative assessments of the effect of different family members’ opposition on women’s intention to use contraceptives in settings where access to FP services is high. The objective of this study is to evaluate the influence of different family members’ opposition to FP on intention to use contraception amongst women living in urban areas who have access to healthcare and FP services, using primary data. The study focused on examining general, long-term intention to use any form of contraception rather than opposition to specific methods of contraception or short-term avoidance of contraception owing to a desire to have more children.

## Methods

An unmatched case control study design was employed. The case control study was nested within a larger prospective cohort study of a novel device to assist with complicated labour, in which eligible women were followed from presentation for delivery until discharge. Full details of the cohort study design and objectives can be found in another paper published by the authors [12]; here we provide a summary of the methods employed to investigate contraceptive intent as a sub-study . All women presenting to the labour rooms of two large government hospitals in Karachi between April and September 2013 with singleton pregnancy and indications of uncomplicated vaginal delivery were eligible to participate. Both hospitals employed female staff to provide information about or direct access to FP services; hence all study participants were aware of the availability of contraceptive services at the hospital. Based on the attending doctors assessment at presentation, women with indications of the following clinical complications (that could result in an emergency transfer to a specialist unit) were excluded: placenta previa, hepatitis B or C, cephalopelvic disproportion (CPD), prolonged rupture of membranes or breech pregnancy.

Eligible women who consented to participate were interviewed by midwives trained in primary data collection. Interviews were conducted 8–24 hours post delivery, when women were alone and waiting to be discharged. Information on women’s intention to use contraception in the future was solicited by first mentioning the local names of a list of 11 contraceptive methods and asking (one by one) if the woman was aware of it (knowledge of contraception assessment) and then asking whether they intend to use any form of contraception in the near or distant future. The purpose of touching on different forms of contraception prior to asking whether the woman intends to use contraception in the future was to make clear that we were interested in intended use of any family planning method in the future. If a woman responded that she would use contraceptive methods once her family was complete but not at present, this was taken to indicate intended use in the future. Information on women’s age, literacy levels, number of previous births, opposition to FP by different family members, opposition by women themselves and knowledge of contraception was collected using structured questionnaires.

An initial cross-sectional analysis was conducted to determine the proportion of women falling into each of the following categories: intend to use contraception; do not intend to use contraception; do not know whether they intend to use contraception or not. Based on the cross-sectional analysis, all women stating that they had no intention to use any forms of contraception at any time in the future, were classified as ‘contraceptive non-intenders’ (cases). ‘Contraceptive intenders’ were defined as women stating that they intend to use contraceptive methods in the near future or after they have reached their ideal family size. Controls were randomly selected from those women that were ‘contraceptive intenders’ such that the number of controls was twice the number of cases.

Univariable and multivariable logistic regression analysis was conducted to compare risk factors between cases and controls; all variables in the univariable analysis were included in the multivariable model. Odds ratios and 95% confidence intervals for each risk factor studied are reported.

Based on data from a large Population Council survey [13] it was estimated that 30% of cases would report opposition to FP by spouses. In order to detect an odds ratio of 2 (that contraceptive non-intenders were twice as likely to report spouse’s opposition as contraceptive intenders), with a control to case ratio of 2:1, a power of 90% and alpha risk of 0.05, the minimum sample size required was 181 cases.

This study received ethical approval from independent Ethical Review Board of HOPE, Pakistan.

## Results

During the study period, 1269 women presented to the study maternity units for vaginal delivery, of which 65 refused to participate in the study and 44 were found to be ineligible owing to indications of potential clinical complications during delivery. Thus, 1160 women were included in the prospective cohort study. The initial cross-sectional analysis showed that 674 women (83%) intended to use contraception in the future, 248 (31%) did not intend to use any contraception in the future, 237 (29%) did not know whether they would use contraceptive methods and one woman refused to answer any questions relating to contraception. Overall 744 women (248 cases and 496 controls) were included in the nested case control study; over 95% had a low literacy level (could not read) and were aged between 16 and 30.

In the univariable analysis Table 1 odds of negative contraceptive intent were lower in women with more than three children (OR = 0.47[0.30-0.73]; p = 0.001) and was higher in women with no knowledge of contraception (OR = 2.63 [1.91-3.65]; p < 0.001), women whose husbands opposed contraception (OR = 18.62 [11.86-29.22]; p < 0.001) and women whose mother in-laws opposed contraception (OR = 3.75 [1.95-7.23]; p < 0.001).

**Table 1:** **Baseline characteristics of contraceptive intenders (cases) and contraceptive intenders (controls) and results of univariable analysis examining risk factors for negative contraception intent**

**Characteristics**	**Number and % of contraceptive non-intenders (n = 248)**	**Number and % of contraceptive intenders (n = 496)**	**Unadjusted odds ratio (95% CI)**	**p-value**
Age				
*16-20*	54 (22%)	71 (14%)	Reference	
*21-25*	112 (45%)	217 (44%)	0.68 (0.45-1.03)	0.071
*26-30*	59 (24%)	159 (32%)	0.49 (0.31-0.78)	0.002
*31-40*	23 (9%)	49 (10%)	0.62 (0.34-1.13)	0.12
No of previous births				
*0*	100 (40%)	157 (32%)	Reference	
*1*	68 (27%)	111 (22%)	0.96 (0.65-1.42)	0.846
*2*	42 (17%)	101 (20%)	0.65 (0.42-1.01)	0.057
*> = 3*	38 (15%)	127 (26%)	0.47 (0.30-0.73)	0.001
Knowledge of contraception				
*One or more methods*	136 (55%)	378 (76%)	Reference	
*No knowledge*	112 (45%)	118 (24%)	2.63 (1.91-3.65)	<0.001
Opposition by family member				
*Yourself (No)*	221 (89%)	495 (99%)	Reference	
*Yourself (Yes)*	27 (11%)	1 (0.2%)	60.47 (8.17-447.84)	<0.001
*Husband (No)*	115 (46%)	467 (94%)	Reference	
*Husband (Yes)*	133 (54%)	29 (6%)	18.62 (11.87-29.22)	<0.001
*Mother-in-law (No)*	222 (90%)	481 (97%)	Reference	
*Mother-in-law (Yes)*	26 (10%)	15 (3%)	3.75 (1.95-7.23)	<0.001
*Mother (No)*	244 (98%)	490 (99%)	Reference	
*Mother (Yes)*	4 (2%)	6 (1%)	1.34 (0.37-4.79)	0.654

In the multivariable analysis Table 2 there was little evidence of an independent effect of parity on contraceptive intent (AOR for three or more previous births = 0.64[0.34-1.21]; p = 0.169). The effect of no knowledge of contraception (AOR = 3.79 [2.43-5.90]; p < 0.001), husband’s opposition to contraception (AOR = 21.87 [13.21-36.2]; p < 0.001) and mother-in-law’s opposition to contraception (AOR = 4.06 [1.77-9.30]; p < 0.001) on contraceptive intent was strong even in the multivariable analysis.

**Table 2:** **Results of multivariate analysis examining risk factors for negative contraception intent**

**Characteristics**	**Adjusted odds ratio (95% CI)**	**p-value**
Age		
*16-20*	Reference	
*21-25*	1.04 (0.60-1.82)	0.887
*26-30*	0.79 (0.42-1.48)	0.456
*31-40*	1.05 (0.44-2.50)	0.905
No of previous births		
*0*		
*1*	1.32 (0.78-2.22)	0.296
*2*	1.21 (0.66-2.21)	0.537
*> = 3*	0.64 (0.34-1.21)	0.169
Knowledge of contraception		
*One or more methods*	Reference	
*No knowledge*	3.79 (2.43-5.90)	<0.001
Opposition by		
*Yourself (No)*	Reference	
*Yourself (Yes)*	23.56 (2.61-212.35)	0.005
*Husband (No)*	Reference	
*Husband (Yes)*	21.87 (13.21-36.21)	<0.001
*Mother-in-law (No)*	Reference	
*Mother-in-law (Yes)*	4.06 (1.77-9.30)	0.001
*Mother (No)*	Reference	
*Mother (Yes)*	1.29 (0.28-5.93)	0.774

Very few women, 27 (11%) of cases and 1 (0.2%) of controls, were opposed to contraception use themselves; odds ratios calculated for this variable are therefore unreliable. Similarly, only a small number of women’s mothers amongst both cases and controls were reported to be opposed to contraception, and perceived opposition by women’s mothers was not associated with negative contraceptive intent (AOR = 1.29 [0.28-5.93]; p = 0.774). Age was not found to be associated with contraceptive intent in either the univariable or multivariable analysis.

## Discussion

This study indicates that perceived opposition to contraception by husbands is the strongest risk factor for negative contraceptive intent among women, even when they have information about and access to FP services. A woman who stated that her husband opposes contraception use was 22 times less likely to intend to use contraception even if she had knowledge, physical access and was not opposed to contraception herself. Other factors such as opposition by mother-in-laws and participant’s knowledge about contraceptive methods were also independently found to increase risk of negative contraception intent (four-fold), but less strongly than husband’s opposition. Of 133 contraceptive non-intenders stating that their husband’s opposed contraception, only 27 said that they also opposed use of contraceptives themselves; this indicates that contraceptive non-intenders held different opinions about contraception than their husbands but based their intentions to use contraception on their husband’s views rather than their own.

A limitation of the study is that we recorded perceived opposition by family members and were not able to interview the concerned family member to confirm if the opposition truly exists. While perceived opposition may ultimately be what influences a woman’s behaviour, further studies investigating whether women correctly gauge family members’ opinions and the influence of communication barriers on women’s perception of opposition would be useful. Similarly, we acknowledge the possibility of bias in women’s responses about their own opposition to contraception due to social pressure; women may have felt social pressure to indicate a lack of interest in contraception or alternatively to report being unopposed to contraception to please the interviewer. To limit reporting bias we ensured that data collectors wore different uniforms from hospital staff and made clear at the beginning of the interview that the questions asked are for research purposes and will not be linked to any information (about contraception) previously provided the hospital staff or with any future treatment received. Another limitation is that we were not able to collect objective information about socio-economic status due to the limited time available to interview women before they were discharged post-delivery. Since the study was conducted in government hospitals providing free services, the participants were all poor, but variations in level of poverty could not be adjusted for in the analysis.

This study is the first to quantitatively investigate the relative influence of perceived opposition by different family members on women’s intention to use contraception. We employed a case–control study design for the final analysis as the initial cross-sectional analysis indicated that 29% of women were not sure about the intention to use contraception in the future and we preferred not to include this subgroup of women in the final analysis. Opposition by family members and other social barriers influencing women’s intention to use contraception have previously been investigated in qualitative studies [6,7]. These results advance the findings from qualitative research by quantitatively indicating how strongly husband’s opposition influences women’s contraceptive intent. The findings are consistent with another quantitative study reporting that choosing one’s own spouse was associated with agreement on number of children and intention to use contraceptives [9]. Our study population was deliberately chosen to include women who were already accessing public hospitals located in the heart of a large urban city and where information on family planning freely available as we aimed for our study to identify factors that are barriers to contraception use even when free health services are available and being accessed. Our results are not therefore representative of the wider population of women including those who are not married, have not had any children or do not use formal maternal and child health services.

## Conclusion

The results of this study indicates that of all family members, husband’s opposition has the strongest effect on women’s intention to use contraception. As our study was conducted in a hospital setting in which FP services were available, it provides evidence that physical access to services and knowledge about contraceptive methods is necessary but not sufficient to ensure intention to use contraception. Since opinions of husband and husband’s families are strong determinants of women’s intention to use contraception in Pakistan, the slow progress on contraception uptake may only improve by employing strategies that effectively generate support for FP among these key stakeholders.

## References

[1] Khan A, Shaikh BT (2013). An all time low utilization of intrauterine contraceptive device as a birth spacing method–a qualitative descriptive study in district Rawalpindi, Pakistan. Reprod Health.

[2] Khan AA, Abbas K, Hamza HB, Bilal A, Khan A (2013). From contraceptive prevalence to family planning service users: implications for policy and programmes. J Pak Med Assoc.

[3] Sultan M, Cleland JG, Ali MM (2002). Assessment of a new approach to family planning services in rural Pakistan. Am J Public Health.

[4] Carton TW, Agha S (2012). Changes in contraceptive use and method mix in Pakistan: 1990–91 to 2006–07. Health Policy Plan.

[5] Sirageldin I, Norris D, Hardee JG (1976). Family planning in Pakistan: an analysis of some factors constraining use. Stud Fam Plan.

[6] Kamran I, Arif MS, Vassos K (2011). Concordance and discordance of couples living in a rural Pakistani village: perspectives on contraception and abortion–a qualitative study. Glob Public Health.

[7] Winkvist A, Akhtar HZ (2000). God should give daughters to rich families only: attitudes towards childbearing among low-income women in Punjab, Pakistan. Soc Sci Med.

[8] Agha S (2010). Intentions to use contraceptives in Pakistan: implications for behavior change campaigns. BMC Public Health.

[9] Hamid S, Stephenson R, Rubenson B (2011). Marriage decision making, spousal communication, and reproductive health among married youth in Pakistan. Glob Health Action.

[10] Shaikh BT (2010). Unmet need for family planning in Pakistan – PDHS 2006–2007: It’s time to re-examine déjà vu. Open Access J Contracept.

[11] Khan AW, Amjad CM, Hafeez A, Shareef R (2012). Perceived individual and community barriers in the provision of family planning services by lady health workers in Tehsil Gujar Khan. J Pak Med Assoc.

[12] Khan M, Hashmani, FN, Ahmed S, Ahsmed O, Asim SS, WajahatY, et al. Prospective cohort study of a new vacuum delivery device to assist with complicated labour in low-resource settings. Trop Med Int Health. 2014; doi:10.1111/tmi.12427. 10.1111/tmi.12427 25367864

[13] Mahmood A. Birth Spacing and Family Planning Uptake in Pakistan: Evidence from FALAH. Population Council Report, 2012.

